# Diabetes Mellitus Affects Urinary Tissue Tropism of Group B Streptococci in a Sex-Dependent Manner

**DOI:** 10.3390/pathogens15090923

**Published:** 2026-09-01

**Authors:** Preeti P. John, Bidhan Gautam, Kenneth A. Rogers, Ritwij Kulkarni

**Affiliations:** 1 School of Biological Sciences, University of Louisiana at Lafayette, Lafayette, LA 70504, USAbidhan.gautam1@louisiana.edu (B.G.); 2 New Iberia Research Center, University of Louisiana at Lafayette, Lafayette, LA 70560, USA

**Keywords:** diabetes mellitus, urinary tract infections, *Streptococcus agalactiae*, group B *Streptococcus*, GBS, GBS-UTI, *db*/*db* mice, sex differences, dissemination

## Abstract

Diabetes mellitus (DM) increases the risk of *Streptococcus agalactiae* (group B *Streptococcus*; GBS) urinary tract infections (UTIs) and severe complications, including pyelonephritis and bacteremia, although underlying molecular mechanisms are unclear. We hypothesized that DM-associated alterations in urinary tract immunity drive GBS-UTI susceptibility. To address this hypothesis, we examined GBS-UTI pathogenesis in *db*/*db* obese hyperglycemic mice. At 24 h post-infection, diabetic (D-) mice showed significantly higher GBS burden in the bladder and the spleen compared to non-diabetic (ND-) controls. This was accompanied by significant dysregulation of the local innate immune response, including reduced leukocyte recruitment and blunted pro-inflammatory cytokine production in both the bladder and kidneys. We also identified sex differences in GBS tissue tropism. D-females showed a higher bladder burden than ND-females, ND-males, and D-males. In contrast, significantly higher kidney burden in ND-males than ND-females was further exacerbated by DM. This correlated with pronounced differences in CXCL-1 levels between male and female bladders in both D and ND cohorts, although sex did not affect immune cell recruitment. Together, these results suggest an association between obese hyperglycemia-mediated increased susceptibility to GBS-UTI and systemic dissemination and impaired local immune responses. In addition, our results also reveal sex-dependent differences in GBS urinary tract colonization.

## 1. Introduction

*Streptococcus agalactiae* (group B *Streptococcus*; GBS), an asymptomatic colonizer of human gastrointestinal and genitourinary tracts, is an atypical uropathogen. It causes an estimated 3–4.5 million urinary tract infections (UTIs) globally, including 160,000 cases per year in the US [1,2,3,4]. Diabetes mellitus (DM) increases the risk of GBS asymptomatic bacteriuria, cystitis, and exacerbations, including pyelonephritis and bloodstream infections (BSI) secondary to UTI (uBSI), according to some [4,5,6,7,8,9,10,11] but not all [12] published reports. The primary objective of this research is to bridge the knowledge gap that the mechanisms underlying the increased susceptibility of diabetic individuals to GBS-UTI have not been fully deciphered.

The diverse effects of the diabetic urinary microenvironment on both host immunity and bacterial virulence are implicated in increasing the risk of UTIs in diabetic individuals. For instance, glycosuria and neurogenic bladder-induced urine retention, the clinical hallmarks of DM, promote growth of uropathogens. Concurrently, DM also weakens immune defenses such as phagocytosis and intracellular killing, rendering them ineffective against these proliferating uropathogens [13,14,15]. We have previously reported that in vitro exposure to glycosuria significantly augments both the growth as well as the virulence of GBS [16]. Interestingly, this response is pathogen-specific, as glycosuria induced virulence of uropathogenic *Escherichia coli* (UPEC) without affecting its growth [17]. Given that GBS utilizes distinct strategies for urinary tract colonization and immune evasion, we decided to examine GBS-UTI pathogenesis in a mouse model of DM.

Patras et al. reported a correlation between higher GBS burden in the urinary bladders of streptozotocin (STZ)-induced diabetic mice and higher antimicrobial peptide cathelicidin (LL-37) levels and increased mast cell activity [18]. Interestingly, both LL-37 and mast cells appear to be ineffective against GBS in diabetic as well as non-diabetic urinary tracts [18]. STZ, a DNA-alkylating glucose analog that destroys endogenous beta cells, resulting in reduced endogenous insulin production and hyperglycemia, is a versatile chemical that can be used to model type 1 DM in a variety of animal models [19]. Indeed, researchers have effectively used the mouse model of STZ-induced DM to study the immunopathology of ascending UTIs by UPEC, *Klebsiella pneumoniae*, *Enterococcus faecalis*, and GBS [18,20,21,22]. However, STZ treatment causes adverse side effects, including nephrotoxicity and immunosuppression (lymphopenia), which can confound the analysis of urinary tract immune responses in DM [23,24]. Moreover, the marked resistance of female mice to the diabetogenic effects of STZ necessitates administration of higher, potentially toxic levels of STZ for inducing DM in female mice [25] making the mouse model of STZ-induced DM unsuitable for studying sex-dependent differences in diabetic UTI pathogenesis. Hence, in this study, we used the *db*/*db* mouse model of genetically induced DM, which models human type 2 DM associated with obesity and hyperglycemia [26]. The increased susceptibility of genetic (*db*/*db*, KK-Ay) models of DM to ascending UPEC-UTI is reported to be associated with reduced inflammation (reduced neutrophil recruitment, delayed IL-6, IL-1β,) as well as impaired insulin signaling leading to reduced urothelial integrity and renal antimicrobial peptide (LCN2, ribonuclease 4) production [27,28,29,30,31,32].

In this study, we inoculated GBS 10/84 via transurethral catheterization into the urinary bladders of 8-week-old *db*/*db* diabetic (D) and non-diabetic (ND) male and female littermates. At 24 h post-infection (hpi), we enumerated GBS CFUs in the urinary tract and spleen. We also characterized local immune response by examining immune cell recruitment and cytokine production in the urinary tract. Our findings demonstrate that obese hyperglycemia enhances susceptibility to GBS-UTI and systemic dissemination while impairing local immune defenses. We also report sex-dependent differences in GBS tissue tropism with DM differentially affecting bladder and kidney colonization in male and female mice.

## 2. Materials and Methods

### 2.1. Model Uropathogen

*Streptococcus agalactiae* CNCTC 10/84 mid-log phase (OD_600_ = 0.6) culture stocks stored in 20% glycerol-sterile tryptic soy broth (TSB) at −20 °C were thawed, washed in sterile phosphate-buffered saline (PBS), resuspended in sterile tryptic soy (TS) broth, and incubated at 37 °C to mid-log phase before use in experiments.

### 2.2. The Murine Model of Ascending UTI in Diabetes

Protocols for mouse husbandry and experimentation were approved by IACUC at UL Lafayette (#2020-8717-025-VLW). The breeding trios of heterozygous mice from BKS.Cg-*Dock7^m^* +/+ *Lepr^db^*/J (Jackson Lab strain #000642, referred to as BKS) were maintained in the vivarium at UL Lafayette to generate the male and female diabetic (*Lepr^db^*^/*db*^, denoted as D) and lean, non-diabetic (ND) littermates, which were used for CFU enumeration, flow cytometry analysis, and ELISA. Mice were genotyped at 5 weeks of age. Mice were weighed, and their blood glucose levels were monitored once a week from 5 weeks of age until infection. In addition, GBS organ burden was also examined in D mice from the B6.BKS(D)-*Lepr^db^*/J background (Jackson Lab strain #000697, referred to as B6), which were received due to an order discrepancy. Although ND-B6 was not included in this order, we used D-B6 mice to examine whether the mouse genetic background affected GBS burden. Six or fewer littermates of either male or female sex were housed in Optimice^®^ cages (Animal Care Systems Inc., Centennial, CO, USA) with access to food and water ad libitum. In total, we used 143 animals in this study (detailed power analysis in Section 2.6).

Prior to infection, mice were anesthetized inside the induction chamber of the SomnoSuite^®^ (Kent Scientific Corp, Torrington, CT, USA) isoflurane vaporizer system. Anesthetized mice were placed on a warming pad with a nose cone delivering low-flow anesthesia during infection. Mice were infected via transurethral catheterization with 10^7^ CFU of GBS10/84 resuspended in 50 μL PBS. After recovery from anesthesia, mice were returned to cages with full access to food and water. Mice were checked at 8 hpi for signs of discomfort or illness (lack of grooming, hunched posture). All the researchers involved in this study have undergone training to reduce pain and distress in laboratory mice and rodents. The institutional protocol for addressing pain and distress in laboratory mice as approved by the veterinarian includes the use of analgesia (subcutaneous administration of 0.1 mg/kg buprenorphine). In our experience, transurethral catheterization is a minimally invasive procedure that does not result in overt signs of pain and distress. Indeed, none of the mice used in this study showed signs of discomfort or illness.

At 24 hpi, mice were euthanized by CO_2_ inhalation wherein 5 or fewer mice were placed in an empty, clean chamber into which CO_2_ was introduced at a low flow rate to displace 30–40% air every minute. CO_2_ flow was maintained for 1 min after animals had stopped breathing. This was followed by cervical dislocation. To avoid cross-contamination between organs, the blood was harvested first by cardiac puncture, followed by the spleen, the most distal organ from the site of GBS inoculation, and the kidneys. The urinary bladder, the site of GBS inoculation, was harvested last. After one animal was dissected, the dissecting tools were cleaned with 95% ethanol and new gloves donned. The organ homogenates in sterile PBS were processed for CFU burden by dilution plating, immune cell recruitment by flow cytometry, or cytokine levels by ELISA. There were no leftover animals at the end of this study.

### 2.3. Bacterial Organ Burden

**Figure 1:**
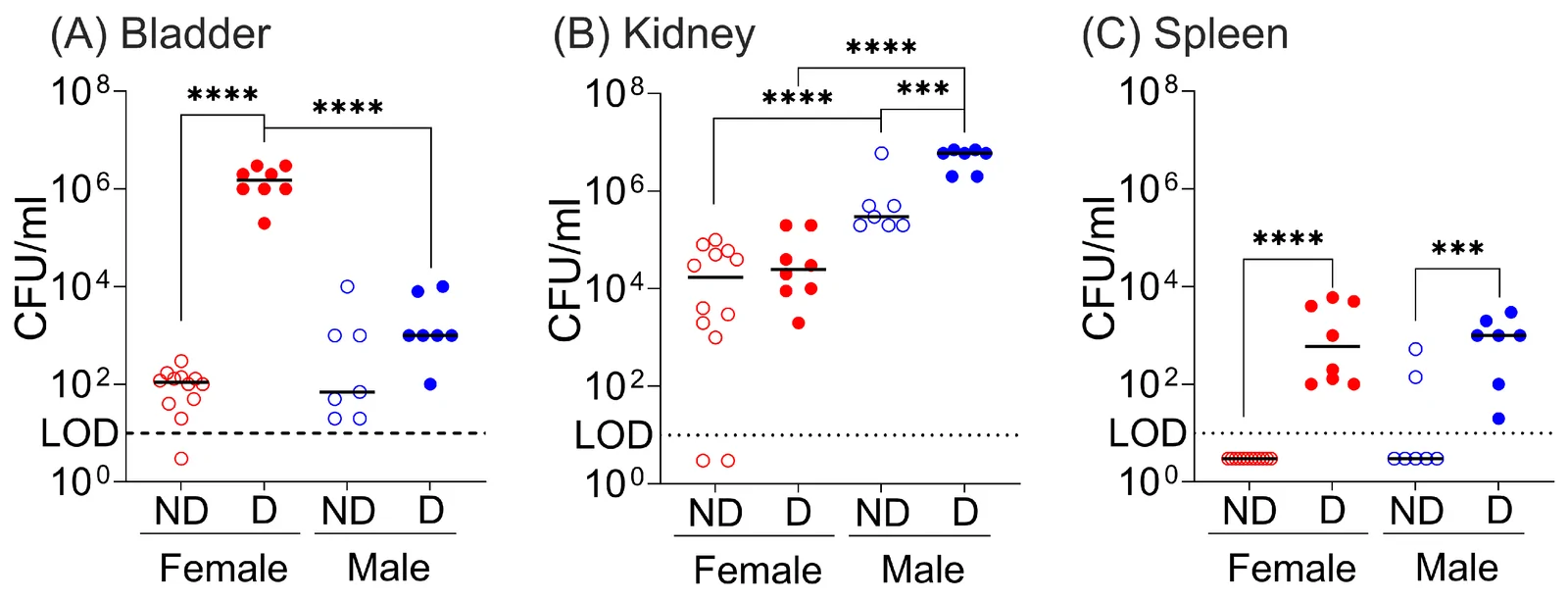
Effects of DM and sex on GBS burden in the urinary tract and the spleen. Eight-week-old female (red) and male (blue), diabetic (D-) and non-diabetic (ND-) littermates from the BKS background were inoculated via transurethral catheterization with GBS 10/84. At 24 hpi, CFU burden in (**A**) the urinary bladder, (**B**) the kidneys, and (**C**) the spleen was determined by dilution plating. Scatter plots show CFU/mL in the organs of an individual mouse with a line indicating the median as the measure of central tendency. The dotted line at 10 CFU/mL indicates the limit of detection (LOD). The data were compared using the Mann–Whitney U test. *** indicates *p* < 0.001, and ****, *p* < 0.0001.

Homogenized bladder, kidney, and spleen tissues from different mouse cohorts collected at 24 hpi were dilution-plated on blood agar plates and incubated overnight at 37 °C. The CFU data in Figure 1 are from 50 mice, including 12 ND-females, 8 D-females, 7 ND-males, and 7 D-males from the BKS background and 8 D-females and 8 D-males from the B6 background. For pairwise comparisons, we used the Mann–Whitney U test, while for factorial comparison, the data were log-transformed and analyzed using two-way ANOVA, as explained in Section 2.6.

### 2.4. Flow Cytometry Analysis

The male and female D and ND littermates from the BKS background were inoculated with either GBS 10/84 or sterile PBS (uninfected control). The flow cytometry analyses were performed on the bladder and kidney tissues collected from infected and control mice at 24 hpi. The analyses included a total of 62 mice, including 32 uninfected (8 each from ND-females, D-females, ND-males, and D-males) and 30 infected (6 ND-females and 8 each from D-females, ND-males, and D-males).

At 24 hpi, whole bladders and right-side kidneys were minced and treated with collagenase IV (10 mg/mL for the bladder and 2 mg/mL for the kidney) and DNase I at room temperature for 90 min while shaking (250 rpm) and frequent pipetting to facilitate dissociating cells. The cell suspension was passed through a 35 μm strainer (Falcon^®^ Thermo Fisher Scientific Inc., Waltham, MA, USA) and washed once in D-PBS (2000 rpm, 5 min, RT). Erythrocytes were lysed with RBC lysis buffer (RT, 10 min) and removed by centrifugation. Next, the cell pellets were stained in tubes protected from light with an amine-reactive viability dye (Alexa Fluor 430 NHS Ester (Succinimidyl Ester, Thermo Fisher Scientific Inc., Waltham, MA, USA) for 25 min at RT. This was followed by surface staining first with 2 μL of Fc block (4 °C for 10 min) and then with a cocktail of fluorescent-conjugated antibodies (2 μL/antibody) against specific leukocyte surface markers (RT for 15 min) [18]. The cell pellets were washed once in FACS buffer (D-PBS + 2% FBS) between staining steps. After the last step, cells were incubated in 250 μL fixation buffer at 4 °C for 20 min, washed once, and resuspended in FACS buffer for use in flow cytometry.

**Figure 2:**
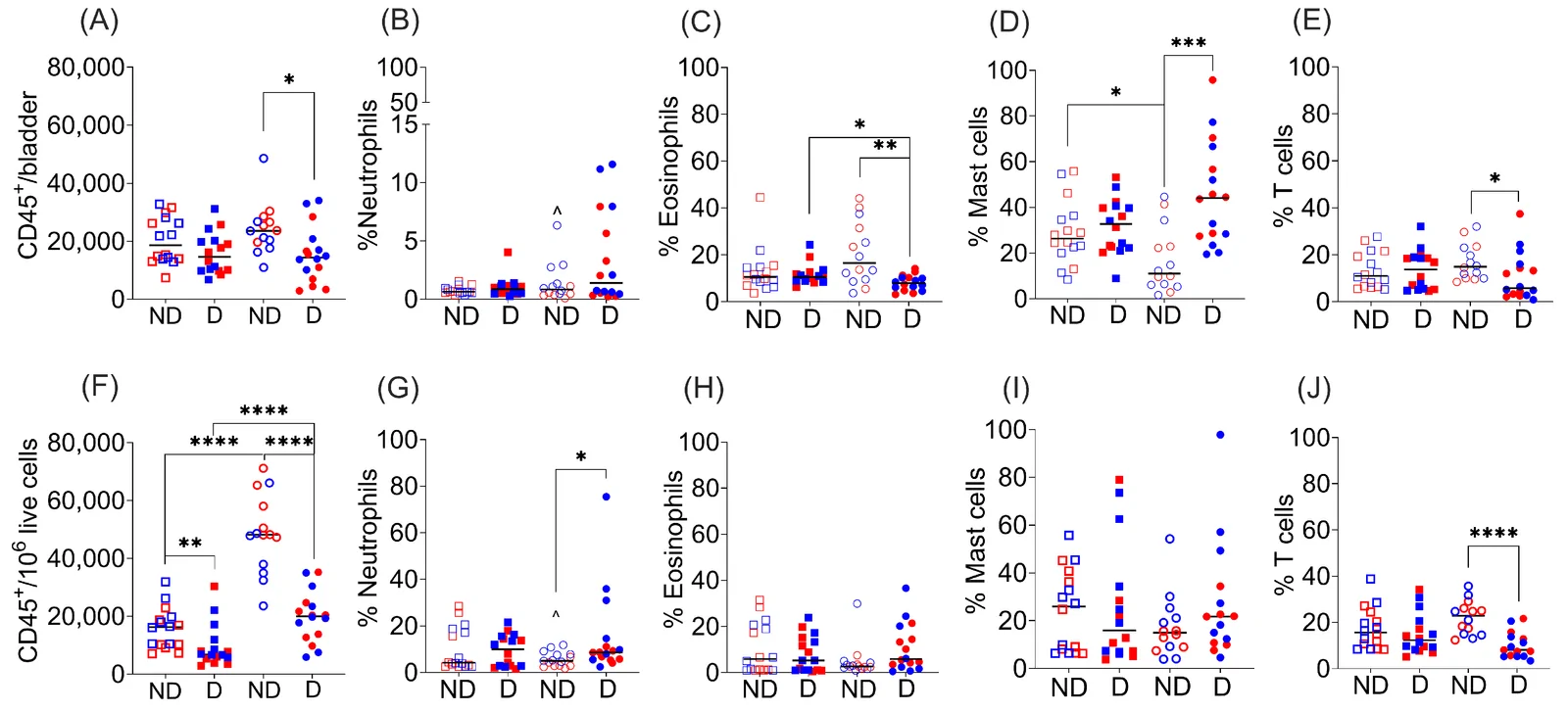
The effects of DM and sex on immune cell recruitment to the GBS-infected urinary tract. At 24 hpi, GBS 10/84 infected (INF) D/ND (closed/open circles), female/male (red/blue) mice or their uninfected (UN) counterparts (closed/open, red/blue squares) were euthanized. Immune cell recruitment to the urinary bladder (panels **A**–**E**) or the kidneys (**F**–**J**) examined by flow cytometry is presented as scatter plots with medians. CD45^+^ total leukocytes are presented as (**A**) total cells/bladder or (**F**) cells/10^6^ live cells in kidney. In contrast, (**B**,**G**) CD45^+^ MHCII^−^ CD11b^+^ Ly6G^+^ neutrophils, (**C**,**H**) CD45^+^ MHCII^−^ CD11b^+^ SiglecF^+^ Ly6G^−^ eosinophils, (**D**,**I**) CD45^+^ CD117^+^ mast cells, and (**E**,**J**) CD45^+^ CD3^+^ T cells are presented as % of CD45^+^ cells. The data were compared using the Mann–Whitney U test. For comparison between uninfected vs. infected, ND vs. D, * indicates *p* < 0.05, **, *p* < 0.01, ***, *p* < 0.001, and ****, *p* < 0.0001. For comparison between male and female mice within a cohort, ^ indicates *p* < 0.05.

The data were analyzed with FlowJO^TM^ version 10. Sequential gating was performed to identify singlets based on FSC-A versus FSC-W, followed by selection of cells based on FSC-A versus SSC-A and exclusion of nonviable cells using the viability dye. CD45^+^ cells were then identified as total leukocytes (Figure S4). Within the CD45^+^ population, MHCII^−^CD11b^+^Ly6G^+^ were regarded as neutrophils, MHCII^−^CD11b^+^SiglecF^+^Ly6G^−^ cells as eosinophils, CD117^+^ cells as mast cells, and CD3^+^ cells as T cells. The scatter plots in Figure 2 show the number of CD45^+^ cells determined from the recovered cell population alongside neutrophil, eosinophil, mast cell, and T cell frequencies as a % of CD45^+^ cells from bladder and kidney tissues of individual mice from uninfected and infected D and ND male and female cohorts. The corresponding raw cell counts are shown in Figure S5. For pairwise comparisons, we used the Mann–Whitney U test, while for factorial comparison, the data were log-transformed and analyzed using three-way ANOVA as explained in Section 2.6.

### 2.5. Multiplex Cytokine ELISA

**Figure 3:**
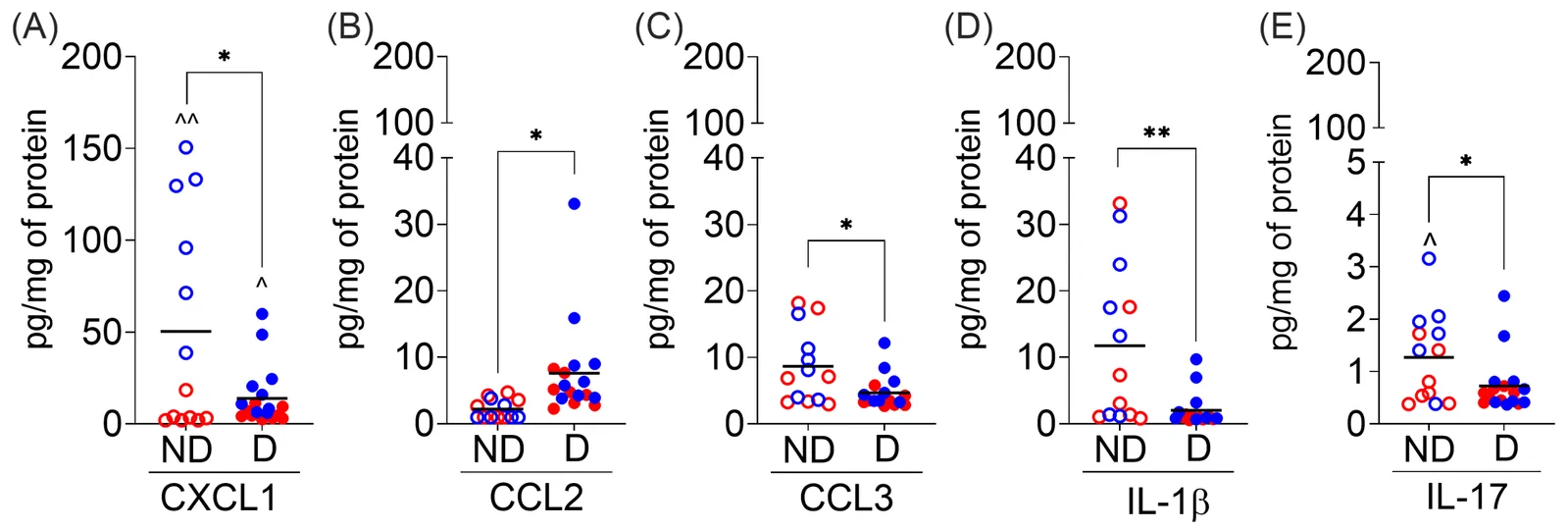
The effects of DM and sex on GBS-mediated cytokine expression in the urinary tract. At 24 hpi, the expression of cytokines such as (**A**) CXCL1 and (**B**) CCL2 in the urinary bladder and (**C**) CCL3, (**D**) IL-1β, and (**E**) IL-17 in the kidneys of GBS 10/84 infected D/ND (closed/open circles), female/male mice (red/blue), was analyzed by multiplex ELISA. Scatter plots show cytokine levels (pg per mg of total protein) in the urinary bladder or the kidneys of an individual mouse with a line indicating the mean. We used an unpaired *t*-test to compare data. For comparison between uninfected/infected, ND/D cohorts (indicated in the x-axis), * indicates *p* < 0.05, and ** indicates *p* < 0.01. For comparison between male and female mice within a cohort, ^ indicates *p* < 0.05 and ^^ indicates *p* < 0.01.

Bladder and kidney homogenates from male, female, D, and ND littermates from the BKS background were filtered through a 0.65 μm Ultrafree^®^-MC Centrifugal Filter (Millipore Sigma, Burlington, MA, USA). The total protein concentration in organ homogenates was estimated using the Pierce BCA protein assay kit (Thermo Fisher Scientific Inc., Waltham, MA, USA). Cytokine levels of IL-1β, IL-6, IL-10, IL-17A, TNF-α, CXCL1 (KC), CCL2 (MCP-1), CCL3 (MIP1α), CCL5 (RANTES), and IFN-γ in whole bladder and kidney tissue homogenates were estimated using MILLIPLEX^®^ Mouse Cytokine/Chemokine Magnetic Bead Panel (MCYTOMAG-70K-10C, Millipore Sigma, Burlington, MA, USA). The ELISA results were obtained from a total of 31 mice, including 7 ND-females, 9 D-females, 6 ND-males, and 9 D-males. The scatter plots in Figure 3 show the amounts of specific cytokines from the bladder or kidney tissues of individual mice and include the means. The ELISA data were treated as parametric. For pairwise comparisons, we used an unpaired *t*-test, while for factorial comparison, the data were log-transformed and analyzed using a three-way ANOVA, as explained in Section 2.6.

### 2.6. Statistical Analysis

A priori power analysis performed at the outset to detect a large biological effect size (Cohen’s *d* = 1.2) with α = 0.05 and 80% statistical power in infection outcomes between D and ND mouse cohorts determined 12 mice per group to be necessary. With a 20% attrition rate, 15 mice/group were initially allocated; 19 ND mice and 31 D mice align with these calculations. However, two unexpected factors altered the final sample size per group: (i) we detected prominent sex-dependent differences in organ burden and decided to explore this by stratifying all datasets by sex, and (ii) a mouse order discrepancy introduced diabetic (*db*/*db*) mice from the B6 background alongside the BKS-background mice. To accommodate these factors, the data were analyzed with multi-factorial tests. The sample sizes—50 mice for CFU (12 ND-females, 8 D-females, 7 ND-males, and 7 D-males from the BKS background and 8 D-females and 8 D-males from the B6 background); 62 for flow cytometry (32 uninfected: 8 each from ND-females, D-females, ND-males, and D-males and 30 infected: 6 ND-females, 8 D-females, 8 ND-males, and 8 D-males); and 31 for ELISA (7 ND-females, 9 D-females, 6 ND-males, and 9 D-males)—were enough to rigorously detect large biological effects of independent variables and their interactions using two-way or three-way ANOVA.

The data were analyzed using GraphPad Prism 10.6.1. Data from multiple biological replicates with two or more technical replicates for each experiment were pooled together. The non-parametric datasets (bacterial organ burden and flow cytometry) are presented as scatter diagrams with medians. The parametric datasets are presented as histograms (mouse weight and blood glucose) or scatter plots (ELISA) with means.

Direct pairwise comparisons between various mouse cohorts—shown as asterisks in figures—were performed independently using Mann–Whitney U tests for non-parametric datasets or *t*-tests for parametric datasets. These pairwise tests were not corrected for multiple comparisons. In addition, to evaluate overall main effects and interactions between independent variables, log-transformed organ burden and ELISA data were analyzed using two-way ANOVA (diabetes × sex), while flow cytometry data were analyzed using three-way ANOVA (diabetes × sex × infection status); post hoc multiple comparisons test was not applied within this analysis. CFUs below the limit of detection (LOD) were assigned a value of 3 CFU/mL prior to statistical analysis. Log transformation was performed as Y = log_10_(Y) prior to two-way ANOVA of organ burden and ELISA data and three-way ANOVA of flow cytometry data. Two-way and three-way ANOVA results are presented as Supplemental Tables. The data were considered statistically significant if *p* ≤ 0.05.

## 3. Results

### 3.1. D-Mice Show Increased Body Weight and Blood Glucose Levels

To address our hypothesis that DM exacerbates GBS-UTI, we experimentally induced ascending UTI by transurethral catheterization of GBS 10/84 into the urinary bladders of 8-week-old male and female *db*/*db* obese, hyperglycemic (diabetic-D), and non-diabetic (ND) mouse littermates. At the time of infection, the average weights of D-females (D = 45.34 ± 5.2 g; ND = 20.5 ± 0.56 g) and D-males (D = 44.97 ± 2.3 g; ND = 23.11 ± 0.99 g) as well as the average blood glucose levels of D-females (D = 391.1 ± 67.1 mg/dL; ND = 148.8 ± 32.5 mg/dL) and D-males (D = 357.9 ± 40 mg/dL; ND = 219.3 ± 39.6 mg/dL) were significantly higher (*p* < 0.0001; Welch’s *t*-test) compared to their ND counterparts (Figure S1).

### 3.2. DM Increases GBS Burden in the Urinary Tract and the Spleen in a Sex-Dependent Manner

At 24 h after experimental induction of GBS ascending UTI, we examined the CFUs in both D and ND littermates from the BKS background as well as in D mice from the B6 background, which we received due to an order discrepancy, as mentioned earlier. Compared to their ND counterparts, the D mice (pooled data from BKS and B6) showed a 2000-fold higher median bladder CFU (D = 2 × 10^5^ CFU/mL; ND = 100 CFU/mL; *p* < 0.0001; Mann–Whitney U test; Figure S2A) and a ~3-fold increase in the median kidney CFU (D = 2 × 10^5^ CFU/mL; ND = 6 × 10^4^ CFU/mL; *p* = 0.064; Figure S2B). In addition, GBS dissemination to the spleens was observed in 31/31 D mice and only 2/19 ND mice (D = 1000 CFU/mL, ND < LOD; *p* < 0.0001; Figure S2C). As GBS was not detected in blood (cardiac puncture), we hypothesize that diabetic mice may develop transient bacteremia at an earlier timepoint.

The D and ND cohorts used in this comparison included both male and female littermates. We subsequently stratified the organ burden data by sex as a post hoc, exploratory analysis. As this was not pre-specified, the cohort sizes after sex-stratification are smaller. In the BKS background mice, DM increased median bladder burden by ~10^4^-fold in females (D-female = 1.5 × 10^6^ CFU/mL; ND-female = 110 CFU/mL; *p* < 0.0001) and by 14-fold in D-males (D-male = 1000 CFU/mL; ND-male = 70 CFU/mL; NS) compared to their ND counterparts; the median bladder GBS burden in ND-female and ND-male cohorts was similar (Figure 1A). In contrast, male sex increased susceptibility to kidney infection by 18-fold over ND mice (ND-male = 3 × 10^5^ CFU/mL; ND-female = 1.7 × 10^4^ CFU/mL; *p* < 0.0001), which was further (~20-fold) exacerbated by the presence of DM (D-male = 6 × 10^6^ CFU/mL; ND-male = 3 × 10^5^ CFU/mL; *p* = 0.047). Conversely, DM did not increase kidney GBS burden in female mice (D-female = 2.5 × 10^4^ CFU/mL; ND-female = 1.7 × 10^4^ CFU/mL; NS) (Figure 1B). Notably, DM-mediated dissemination of GBS to the spleen appears to be sex-independent, as both D-male and D-female mice exhibited significantly higher median CFUs (D-female = 2000 CFU/mL, D-male = 1000 CFU/mL) than their ND counterparts with organ burden below the LOD. GBS CFUs were above the limit of detection in 2/7 ND-male and 0/12 ND-female mouse spleens (Figure 1C). Notably, the data spread as well as median CFU for D-male and D-female mice from the B6 background were similar to their BKS counterparts across the urinary bladder, kidneys, and spleen (Figure S3), suggesting that the mouse genetic background does not affect GBS organ burden.

Two-way ANOVA of log-transformed organ burden data from the BKS mice (Table S1) revealed (i) both DM and sex as significant independent variables with significant interactions in the urinary bladder, (ii) both DM and sex as significant independent variables without significant interaction in the kidney, and (iii) only DM as a significant independent variable in the spleen. Overall, the organ burden results demonstrate that DM increases susceptibility to GBS-UTI and systemic dissemination with GBS tropism to the bladder and kidneys occurring in a sex-dependent manner.

### 3.3. The Effects of DM and Sex on Immune Cell Recruitment to GBS-Infected Urinary Tract

We performed differential flow cytometry analysis of bladder and kidney tissues from uninfected/infected, D/ND, male/female mice. The flow cytometry data are presented in Figure 2 as scatter plots showing the total number of CD45^+^ total leukocytes from each animal or the % of CD45^+^ total leukocytes for neutrophils (CD45^+^MHCII^−^CD11b^+^Ly6G^+^), eosinophils (CD45^+^MHCII^−^CD11b^+^SiglecF^+^Ly6G^−^), mast cells (CD45^+^CD117^+^), and T cells (CD45^+^CD3^+^). The gating scheme for flow cytometry analysis is presented in Figure S4. Raw numbers of immune cells are also presented as scatter plots in Figure S5.

At 24 hpi, GBS bladder infection modestly increased total leukocyte recruitment in ND mice compared to uninfected ND mice. Moreover, DM significantly suppressed the recruitment of CD45^+^ total leukocytes (*p* = 0.0104; Figure 2A), eosinophils (*p* = 0.003; Figure 2C), and T cells (*p* = 0.034; Figure 2D) in response to GBS bladder infection. In contrast, GBS infection significantly (~3-fold) increased recruitment of CD45^+^ total leukocytes to kidneys (*p* < 0.0001 for ND-infected vs. ND-infected) while DM suppressed it by ~3-fold (*p* < 0.0001 for ND-infected vs. D-infected; Figure 2F). Notably, DM increased neutrophil recruitment by ~2-fold (*p* < 0.0001, Figure 2G) while reducing T cell recruitment by ~3-fold (*p* < 0.0001, Figure 2J) to GBS-infected kidneys. When stratified for sex, GBS-infected ND-males showed a modest but significant increase in the median neutrophil counts compared to GBS-infected ND-female bladder (Figure 2B) and kidneys (Figure 2G). Three-way ANOVA (Table S2) revealed (i) DM as a significant independent variable affecting CD45^+^ total leukocytes, neutrophils, eosinophils, and mast cells’ recruitment to the bladder; and (ii) DM and infection as significant independent variables with significant interaction in affecting recruitment of CD45^+^ leukocytes to kidneys.

### 3.4. The Effects of DM and Sex on Cytokine Production in the GBS-Infected UT

Next, we used multiplex ELISA to examine the levels of cytokines (IL-1β, IL-6, IL-10, IL-17, CXCL1, CCL2, CCL3, CCL5, TNFα, and IFNγ) in bladder and kidney homogenates from GBS-infected male and female D and ND littermates. Compared to their ND counterparts, GBS-infected D-bladders produced significantly lower CXCL1 (*p* = 0.0165, unpaired *t*-test; Figure 3A) and significantly higher CCL2 (*p* = 0.0234; Figure 3B). Bladder CXCL1 levels were also significantly higher in both D (*p* = 0.0318 ) and ND (*p* = 0.0023) males compared to their female counterparts (Figure 3A). Compared to their ND counterparts, GBS-infected D-kidneys produced significantly lower levels of CCL3 (*p* = 0.0172; Figure 3C), IL-1β (*p* = 0.0037; Figure 3D), and IL-17 (*p* = 0.0413; Figure 3E). Notably, ND-males produced significantly higher kidney IL-17 compared to ND-females in response to GBS-UTI (*p* = 0.039; Figure 3E). Cytokines unaffected by either DM or sex are not shown. Overall, the ELISA results indicate that diabetes dampens GBS-induced proinflammatory cytokine (CXCL1, CCL3, IL-1β, IL-17) production in the urinary tract. Two-way ANOVA (Table S3) of ELISA data revealed (i) both DM and sex as significant independent variables with significant interaction affecting CXCL1 production in the bladder, (ii) DM as a significant variable affecting bladder CCL2 and kidney CCL3 and IL-1β, and (iii) both DM and sex as significant variables affecting kidney IL-17 without significant interaction.

## 4. Discussion

DM is a major risk factor for UTIs and their exacerbations, including pyelonephritis and uBSI [33]. In this study, we examined the effects of DM on the pathophysiology of GBS-UTI. The post hoc stratification of the data into male and female mouse cohorts facilitated, quite serendipitously, the analysis of sexual dimorphism in GBS urinary tissue tropism. As the model uropathogen, we used GBS 10/84, a clinical isolate that belongs to serotype V, one of the three predominant GBS serotypes known to cause UTI in humans [12]. Given that GBS 10/84 is hyperhemolytic [34], it is important that future studies examine the pathogenesis of diabetic UTIs using GBS clinical isolates from UTI patients. We inoculated GBS 10/84 via transurethral catheterization to induce ascending UTI in the *db*/*db* mouse model of genetically induced DM. Due to a homozygous spontaneous mutation in *Lepr* (leptin receptor), the *db*/*db* mice exhibit polyphagia, which, in turn, results in obesity, hyperglycemia, and glycosuria around 4–6 weeks of age [35]. For our experiments, we bred mice heterozygous for the *Lepr* mutation (*db*/+) to generate diabetic (D-*db*/*db* homozygotes) and non-diabetic (ND-*db*/+ or +/+) littermates. The F2-generation littermates are considered a gold standard for standardizing gut microbiota in experimental animals as they experience the same microbial and environmental exposures up to and following birth [36].

At 24 hpi, compared to their ND counterparts, the *db*/*db* mouse bladders and spleens exhibited significantly higher GBS burden, while the *db*/*db* kidneys showed a substantial yet statistically insignificant increase in GBS CFUs. The inclusion of both male and female littermates allowed us to stratify the organ burden data to reveal that female D mice were more susceptible to GBS cystitis. Moreover, GBS preferentially colonized the kidneys across male and female, D and ND mouse cohorts, which aligns with previous reports examining urinary pathogenesis of various GBS strains, including 10/84 [16,37,38]. However, it is novel that males exhibited higher susceptibility to GBS kidney infection than females and the presence of DM increased the susceptibility to kidney infection even further. Both male and female D mice were equally susceptible to GBS dissemination. In addition to establishing DM as a risk factor for GBS-UTI, our observations revealed a novel and major role for DM in promoting GBS dissemination from the urinary tract to spleen at 24 hpi and a novel sex-specific UT tissue tropism. A high kidney burden in ND-males without a correspondingly high spleen burden suggests that ND kidneys may possess a barrier restricting the systemic spread of GBS, which may be impaired by DM.

In comparison to their ND counterparts, the D-bladder and D-kidney tissues also showed lower pro-inflammatory cytokine production and reduced CD45^+^ leukocyte recruitment in response to GBS infection. Moreover, compared to their female counterparts, both D- and ND-male bladders infected with GBS showed significantly higher levels of CXCL1, a neutrophil chemoattractant cytokine, while increased neutrophil recruitment was noted in the bladder as well as kidneys of ND-males with GBS infection. These novel and significant observations do not confirm a causal relationship between immune dysfunction and GBS organ burden. Future experiments where specific immune effectors are ablated in male and female D and ND mouse cohorts prior to the induction of GBS ascending UTIs may reveal mechanistic underpinnings of increased susceptibility and tissue tropism in the pathogenesis of GBS diabetic UTIs.

In 2020, Patras et al. reported increased susceptibility to GBS-UTI in the mouse model of STZ-induced DM [18]. Our study complements this seminal report by providing insights into the role of DM in promoting GBS dissemination to the spleen and by revealing sex-dependent differences in GBS-UTI pathogenesis. Moreover, compared to their ND counterparts, both *db*/*db* and STZ mice show significantly higher bladder GBS burden and higher mast cell recruitment to the bladder in response to GBS infection. The differences between the results from STZ and *db*/*db* mice, such as the substantial difference in the magnitude of increment in bladder CFUs (1.13-fold for STZ and 2000-fold for *db*/*db*) and different effects of DM on kidney CFUs (DM reduced kidney CFUs in STZ mice while increasing them in *db*/*db* mice), may be likely due to the differences in the mouse models of DM (chemically induced versus genetic origin) or GBS strains (COH1 versus 10/84).

Our ongoing time course experiments comparing GBS-UTI pathogenesis between D and ND littermates at various early (6, 10, and 12 hpi) and late (days 7, 14, and 28 pi) time points aim to address various acknowledged limitations of this study. For example, (i) blood CFU analysis at early time points will help us determine whether DM promotes transient bacteremia secondary to GBS-UTI early in the infection and may explain why we detected GBS CFUs only in the spleens and not in the blood of all D mice at 24 hpi; (ii) flow cytometry and ELISA of GBS-infected bladder and kidney tissues from male/female D/ND mice at 6 hpi will identify immune effectors differentially affected early in the infection by DM and/or sex; and (iii) comparing GBS-UTI in male/female D/ND mice at days 7, 14, and 28 pi will help us assess the effects of DM and/or sex on chronicity and severity of GBS-UTI complications. Additional future experiments include in vivo imaging to track bioluminescent bacteria to determine whether GBS ascends to the kidneys via ureters or via hematogenous or lymphatic routes; the examination of the urinary pathogenesis of GBS clinical isolates from human UTI patients; and exploration of the differential contributions of obesity, hyperglycemia, and sex to the pathogenesis of UTI caused by different uropathogens, including GBS.

## Data Availability

Raw flow cytometry and ELISA reads will be made available upon request.

## Supplementary Materials

The following supporting information can be downloaded at https://www.mdpi.com/article/10.3390/pathogens15090923/s1, Figure S1: Mouse weights and blood glucose levels. Mean weights (A) and blood glucose levels (B) of male (blue) and female (red), diabetic (D) and non-diabetic littermates measured on the day of infection. Standard deviations are shown as error bars. Data were compared with unpaired t test with Welch’s correction. **** indicates *p* < 0.0001; Figure S2: Effects of DM on GBS organ burden. CFU data from both male and female littermates within obese, hyperglycemic (D, closed circles) or lean, normoglycemic (ND, open circles) cohorts at 24 hpi are shown for (A) the urinary bladder, (B) the kidneys, and (C) the spleen. The scatter plots with median include pooled data from BKS and B6 background mice. The dotted line indicates the limit of detection (LOD) at 10 CFU/mL. **** indicates *p* < 0.0001 for pairwise comparison by Mann-Whitney U test.; Figure S3: Comparison of GBS organ burden between D mice from different genetic backgrounds. CFU from female (F) and male (M) D-mice from BKS and B6 genetic backgrounds were enumerated at 24 hpi. The CFU data from (A) the urinary bladder, (B) the kidneys, and (C) the spleen are shown as scatter plots with median. The dotted line indicates the limit of detection (LOD) at 10 CFU/mL. *** indicates *p* < 0.001 for pairwise comparison by the Mann-Whitney U test; Figure S4: Flow cytometry gating scheme. Using FlowJO^TM^ version 10, sequential gating was performed in the following order- Singlets → FSC-A/SSC-A → viability exclusion → CD45^+^ → marker-defined subsets. Note: monocyte-derived cells, resolved from neutrophils and eosinophils by SiglecF/Ly6G staining, were not further characterized in this study.; Figure S5: Raw flow cytometry data. Raw numbers of immune cells in (panels A–E) the urinary bladder and (panels F–J) the kidney of PBS-inoculated uninfected controls (rectangles) and GBS-infected (circles), non-diabetic (ND, open symbols) and diabetic (D, closed symbols), female (red) and male (blue) mouse cohorts are shown as scatter plots with median. Total number of (A,F) CD45^+^ total leukocytes, (B,G) neutrophils, (C,H) eosinophils, (D,I) mast cells, and (E,J) T cells are shown either as cells/bladder (A–E) or cells/106 live kidney cells (F–J). Pairwise comparison was done using the Mann-Whitney U test. * indicates *p* < 0.05, **, *p* < 0.01, ***, *p* < 0.001, and ****, *p* < 0.0001 for comparison between uninfected and infected, ND and D cohorts, ^ indicates *p* < 0.05 for male and female comparison.; Table S1: Two-way ANOVA results for log-transformed organ burden data. Rows are bolded where *p* < 0.05; Table S2: Three-way ANOVA results for flow cytometry data. Rows are bolded where *p* < 0.05; Table S3: Two-way ANOVA results for ELISA data. Rows are bolded where *p* < 0.05.

## Abbreviations

The following abbreviations are used in this manuscript:

UTIs	Urinary tract infections
DM	Diabetes mellitus
GBS	Group B *Streptococcus*
CFU	Colony-forming units
