# Supplementary material for: Diabetes Mellitus Affects Urinary Tissue Tropism of Group B Streptococci in a Sex-Dependent Manner
Source: Pathogens. 2026 Sep 1;15(9):923. doi: 10.3390/pathogens15090923 (PMC13610892; doi:10.3390/pathogens15090923)
Supplement: Supplementary file 1 [file pathogens-15-00923-s001.zip › pathogens-4407734-supplementary final 2 - update/Figure S1.pdf]

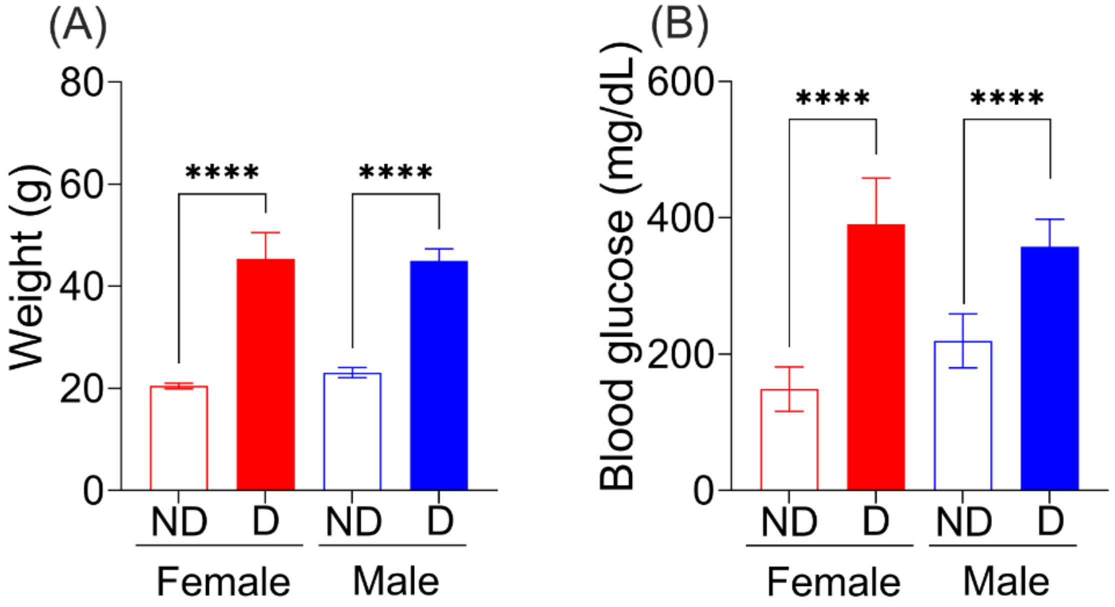

Figure S1: Mouse weights and blood glucose levels. Mean weights (A) and blood glucose levels (B) of male (blue) and female (red), diabetic (D) and non-diabetic littermates measured on the day of infection. Standard deviations are shown as error bars. Data were compared with unpaired t test with Welch's correction. **** indicates $p < 0.0001$
