# Supplementary material for: Diabetes Mellitus Affects Urinary Tissue Tropism of Group B Streptococci in a Sex-Dependent Manner
Source: Pathogens. 2026 Sep 1;15(9):923. doi: 10.3390/pathogens15090923 (PMC13610892; doi:10.3390/pathogens15090923)
Supplement: Supplementary file 1 [file pathogens-15-00923-s001.zip › pathogens-4407734-supplementary final 2 - update/Figure S2 CFU D v ND Data.pdf]

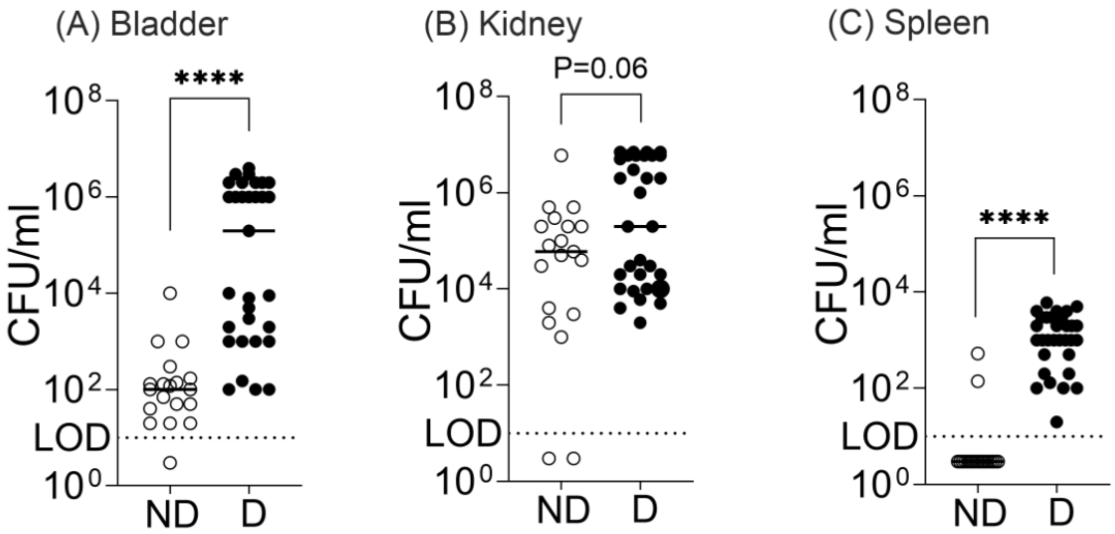

Figure S2: Effects of DM on GBS organ burden. CFU data from both male and female littermates within obese, hyperglycemic (D, closed circles) or lean, normoglycemic (ND, open circles) cohorts at 24 hpi are shown for (A) the urinary bladder, (B) the kidneys, and (C) the spleen. The scatter plots with median include pooled data from BKS and B6 background mice. The dotted line indicates the limit of detection (LOD) at 10 CFU/ml. **** indicates P<0.0001 for pairwise comparison by Mann-Whitney U test.
