# Supplementary material for: Diabetes Mellitus Affects Urinary Tissue Tropism of Group B Streptococci in a Sex-Dependent Manner
Source: Pathogens. 2026 Sep 1;15(9):923. doi: 10.3390/pathogens15090923 (PMC13610892; doi:10.3390/pathogens15090923)
Supplement: Supplementary file 1 [file pathogens-15-00923-s001.zip › pathogens-4407734-supplementary final 2 - update/Figure S3 BKS v B6 CFU.pdf]

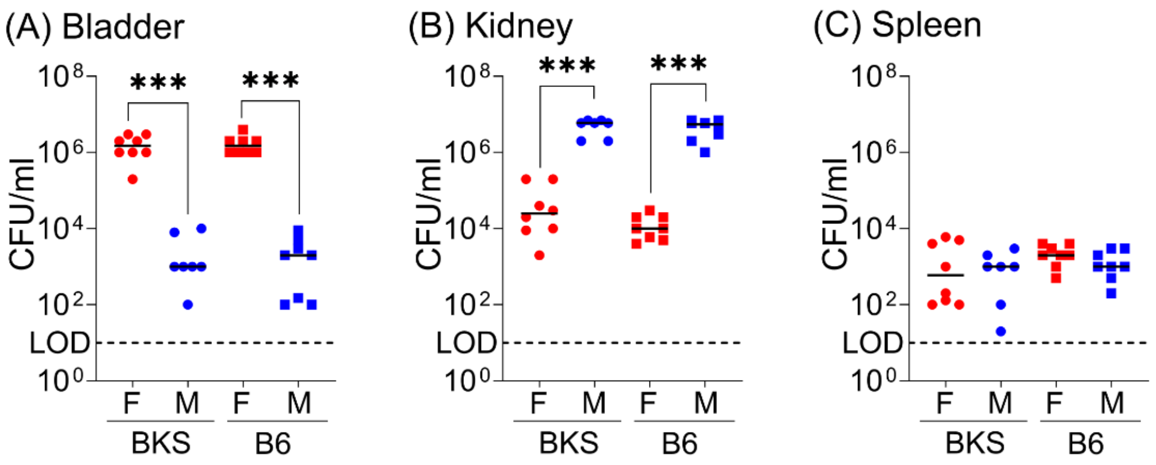

Figure S3: Comparison of GBS organ burden between D mice from different genetic backgrounds. CFU from female (F) and male (M) D -mice from BKS and B6 genetic backgrounds were enumerated at 24 hpi. The CFU data from (A)the urinary bladder, (B)the kidneys, and (C) the spleen are shown as scatter plots with median. The dotted line indicates the limit of detection (LOD) at 10 CFU/ml. *** indicates $p < 0.001$ for pairwise comparison by the Mann-Whitney U test
