# Supplementary material for: Diabetes Mellitus Affects Urinary Tissue Tropism of Group B Streptococci in a Sex-Dependent Manner
Source: Pathogens. 2026 Sep 1;15(9):923. doi: 10.3390/pathogens15090923 (PMC13610892; doi:10.3390/pathogens15090923)
Supplement: Supplementary file 1 [file pathogens-15-00923-s001.zip › pathogens-4407734-supplementary final 2 - update/Figure S4.pdf]

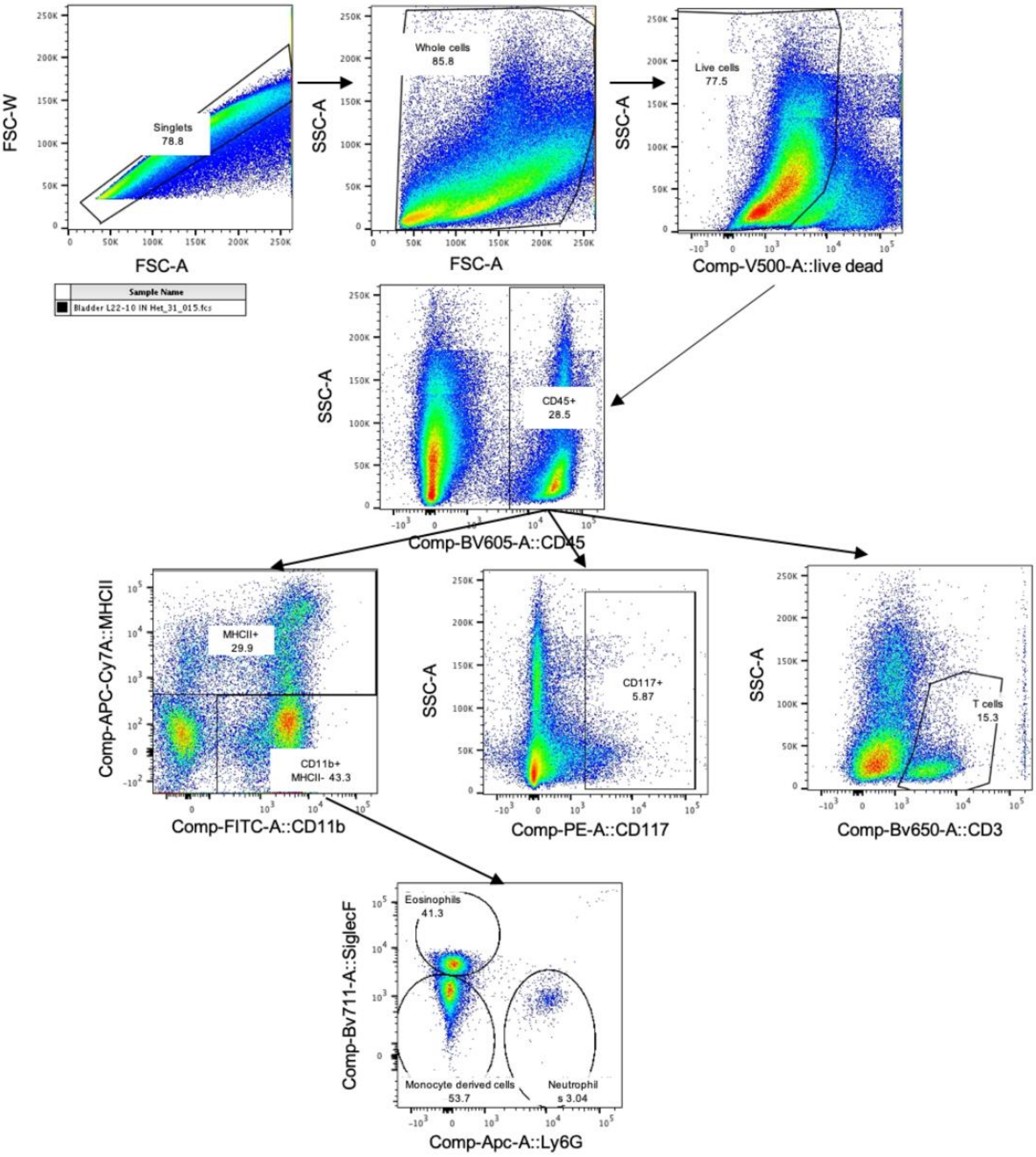

Figure S4: Flow cytometry gating scheme. Using FlowJO™ version 10, sequential gating was performed in the following order- Singlets → FSC-A/SSC-A → viability exclusion → CD45$^+$ → marker-defined subsets. Note: monocyte-derived cells, resolved from neutrophils and eosinophils by SiglecF/Ly6G staining, were not further characterized in this study
