# Supplementary material for: Diabetes Mellitus Affects Urinary Tissue Tropism of Group B Streptococci in a Sex-Dependent Manner
Source: Pathogens. 2026 Sep 1;15(9):923. doi: 10.3390/pathogens15090923 (PMC13610892; doi:10.3390/pathogens15090923)
Supplement: Supplementary file 1 [file pathogens-15-00923-s001.zip › pathogens-4407734-supplementary final 2 - update/Figure S5.pdf]

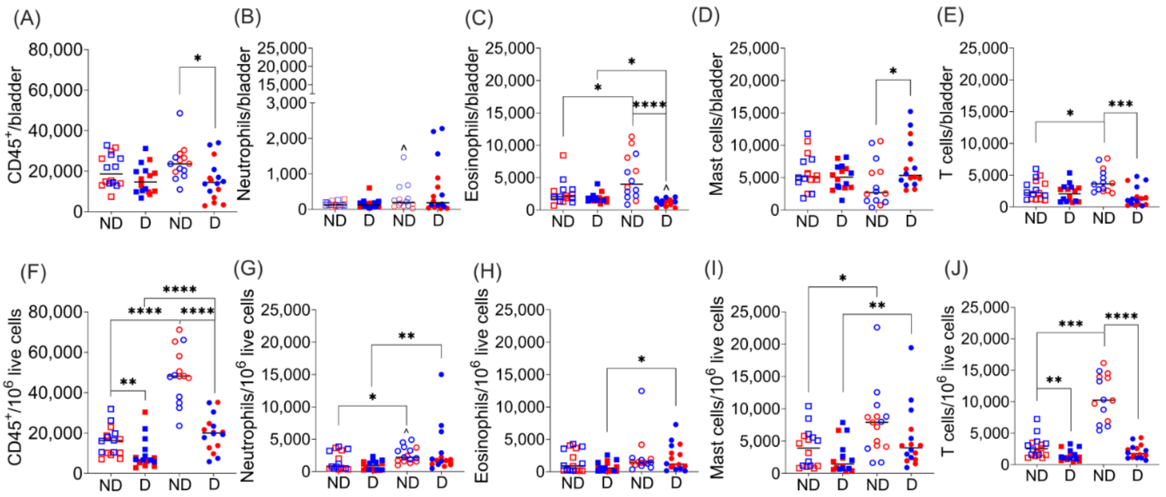

Figure S5: Raw flow cytometry data. Raw numbers of immune cells in (panels A-E) the urinary bladder and (panels F-J) the kidney of PBS-inoculated uninfected controls (rectangles) and GBS-infected (circles), non-diabetic (ND, open symbols) and diabetic (D, closed symbols), female (red) and male (blue) mouse cohorts are shown as scatter plots with median. Total number of (A, F) CD45+ total leukocytes, (B, G) neutrophils, (C, H) eosinophils, (D, I) mast cells, and (E, J) T cells are shown either as cells/bladder (A-E) or cells/106 live kidney cells (F-J). Pairwise comparison was done using the Mann-Whitney U test. * indicates $p < 0.05$, **, $p < 0.01$, ***, $p < 0.001$, and ****, $p < 0.0001$ for comparison between uninfected and infected, ND and D cohorts, ^ indicates $p < 0.05$ for male and female comparison
