# Supplementary material for: Diabetes Mellitus Affects Urinary Tissue Tropism of Group B Streptococci in a Sex-Dependent Manner
Source: Pathogens. 2026 Sep 1;15(9):923. doi: 10.3390/pathogens15090923 (PMC13610892; doi:10.3390/pathogens15090923)
Supplement: Supplementary file 1 [file pathogens-15-00923-s001.zip › pathogens-4407734-supplementary final 2 - update/Table S1.pdf]

	Source of Variation	% of Total Variation	DF	MS	F (DFn, DFd)	P value
**Bladder CFU**	**Diabetes**	**48.16**	**1**	**52.16**	**F (1, 30) = 117.7**	**<0.0001**
	**Sex**	**11.92**	**1**	**12.9**	**F (1, 30) = 29.11**	**<0.0001**
	**Interaction**	**22.01**	**1**	**23.83**	**F (1, 30) = 53.76**	**<0.0001**
	Residual		30	0.4433		
**Kidney CFU**	**Diabetes**	**8.428**	**1**	**6.917**	**F (1, 30) = 6.237**	**0.0182**
	**Sex**	**46.48**	**1**	**38.14**	**F (1, 30) = 34.39**	**<0.0001**
	Interaction	0.07256	1	0.05955	F (1, 30) = 0.053[illegible]	0.8183
	Residual		30	1.109		
**Spleen CFU**	**Diabetes**	**63.34**	**1**	**32.77**	**F (1, 30) = 71.40**	**<0.0001**
	Sex	0.8763	1	0.4534	F (1, 30) = 0.987[illegible]	0.3282
	Interaction	1.631	1	0.8437	F (1, 30) = 1.838	0.1853
	Residual		30	0.459		

Table S1: Two-way ANOVA results for log-transformed organ burden data. Rows are bolded where $P<0.05$.
