# Supplementary material for: Diabetes Mellitus Affects Urinary Tissue Tropism of Group B Streptococci in a Sex-Dependent Manner
Source: Pathogens. 2026 Sep 1;15(9):923. doi: 10.3390/pathogens15090923 (PMC13610892; doi:10.3390/pathogens15090923)
Supplement: Supplementary file 1 [file pathogens-15-00923-s001.zip › pathogens-4407734-supplementary final 2 - update/Table S2.pdf]

	Source of Variation	% of Total Variation	DF	MS	F(DFn, DFd)	P value
**Bladder CD45**	**Diabetes**	**14.24**	1	694857141	F (1, 54) = 9.996	**0.0026**
	Sex	2.066	1	100811626	F (1, 54) = 1.450	0.2337
	Infection	1.078	1	52572560	F (1, 54) = 0.7563	0.3883
	Diabetes x Sex	2.051	1	100049017	F (1, 54) = 1.439	0.2355
	Diabetes x Infection	1.668	1	81395037	F (1, 54) = 1.171	0.284
	Sex x Infection	0.06353	1	3099322	F (1, 54) = 0.04459	0.8336
	Diabetes x Sex x Infection	2.792	1	136218961	F (1, 54) = 1.960	0.1673
	Residual		54	69513454		
**Bladder Neutrophils**	**Diabetes**	**6.31**	**1**	**23.2**	**F (1, 54) = 4.617**	**0.0362**
	Sex	2.349	1	8.637	F (1, 54) = 1.719	0.1954
	**Infection**	**9.297**	**1**	**34.18**	**F (1, 54) = 6.802**	**0.0117**
	Diabetes x Sex	2.55E-05	1	0.00009389	F (1, 54) = 1.868e-005	0.9966
	Diabetes x Infection	3.832	1	14.09	F (1, 54) = 2.804	0.0998
	Sex x Infection	3.985	1	14.65	F (1, 54) = 2.916	0.0934
	Diabetes x Sex x Infection	0.07748	1	0.2849	F (1, 54) = 0.05669	0.8127
	Residual		54	5.025		
**Bladder Eosinophils**	**Diabetes**	**15.22**	**1**	**817**	**F (1, 54) = 11.80**	**0.0011**
	Sex	3.077	1	165.1	F (1, 54) = 2.384	0.1284
	Infection	1.753	1	94.08	F (1, 54) = 1.359	0.2489
	Diabetes x Sex	3.34	1	179.2	F (1, 54) = 2.588	0.1135
	**Diabetes x Infection**	**10.04**	**1**	**538.8**	**F (1, 54) = 7.780**	**0.0073**
	Sex x Infection	1.341	1	71.94	F (1, 54) = 1.039	0.3126
	Diabetes x Sex x Infection	0.5216	1	27.99	F (1, 54) = 0.4042	0.5276
	Residual		54	69.25		
**Bladder Mast Cells**	**Diabetes**	**17.82**	**1**	**3886**	**F (1, 54) = 14.31**	**0.0004**
	Sex	2.255	1	491.7	F (1, 54) = 1.810	0.1841
	Infection	0.07429	1	16.2	F (1, 54) = 0.05963	0.808
	Diabetes x Sex	0.2676	1	58.35	F (1, 54) = 0.2148	0.6449
	**Diabetes x Infection**	**11.86**	**1**	**2586**	**F (1, 54) = 9.521**	**0.0032**
	Sex x Infection	0.1262	1	27.5	F (1, 54) = 0.1013	0.7515
	Diabetes x Sex x Infection	0.477	1	104	F (1, 54) = 0.3829	0.5386
	Residual		54	271.6		
**Bladder T Cells**	Diabetes	3.011	**1**	135.1	F (1, 54) = 1.749	0.1916
	Sex	0.2719	1	12.2	F (1, 54) = 0.1580	0.6926
	Infection	0.03009	1	1.35	F (1, 54) = 0.01748	0.8953
	Diabetes x Sex	0.07479	1	3.357	F (1, 54) = 0.04345	0.8357
	Diabetes x Infection	3.304	**1**	148.3	F (1, 54) = 1.919	0.1716
	Sex x Infection	0.09172	1	4.116	F (1, 54) = 0.05328	0.8183
	Diabetes x Sex x Infection	0.2296	1	10.3	F (1, 54) = 0.1334	0.7164
	Residual		54	77.25		
**Kidney CD45**	**Diabetes**	**25.9**	**1**	**4706407305**	**F (1, 54) = 59.00**	**<0.0001**
	Sex	0.1735	1	31534976	F (1, 54) = 0.3953	0.5322
	**Infection**	**39.47**	**1**	**7171733208**	**F (1, 54) = 89.90**	**<0.0001**
	Diabetes x Sex	0.5682	1	103258808	F (1, 54) = 1.294	0.2603
	**Diabetes x Infection**	**10.81**	**1**	**1964879605**	**F (1, 54) = 24.63**	**<0.0001**
	**Sex x Infection**	**2.564**	**1**	**465973096**	**F (1, 54) = 5.841**	**0.0191**
	Diabetes x Sex x Infection	1.625	1	295250508	F (1, 54) = 3.701	0.0597
	Residual		54	79772633		
**Kidney Neutrophils**	Diabetes	3.96	1	315.7	F (1, 55) = 2.793	0.1003
	Sex	5.569	1	444	F (1, 55) = 3.928	0.0525
	Infection	0.06102	1	4.865	F (1, 55) = 0.04304	0.8364
	Diabetes x Sex	2.806	1	223.7	F (1, 55) = 1.979	0.1651
	Diabetes x Infection	5.104	1	406.9	F (1, 55) = 3.600	0.063
	Sex x Infection	4.086	1	325.7	F (1, 55) = 2.882	0.0952
	Diabetes x Sex x Infection	0.6099	1	48.63	F (1, 55) = 0.4302	0.5146
	Residual		55	113		
**Kidney Eosinophils**	Diabetes	0.8809	1	47.31	F (1, 55) = 0.5223	0.4729
	Sex	0.3917	1	21.04	F (1, 55) = 0.2323	0.6318
	Infection	0.6423	1	34.49	F (1, 55) = 0.3808	0.5397
	Diabetes x Sex	0.03338	1	1.793	F (1, 55) = 0.01979	0.8886
	Diabetes x Infection	4.948	1	265.7	F (1, 55) = 2.934	0.0924
	Sex x Infection	0.4979	1	26.74	F (1, 55) = 0.2953	0.5891
	Diabetes x Sex x Infection	0.2154	1	11.57	F (1, 55) = 0.1277	0.7222
	Residual		55	90.57		
**Kidney Mast Cells**	Diabetes	1.799	1	452.4	F (1, 53) = 1.057	0.3086
	Sex	5.408	1	1360	F (1, 53) = 3.178	0.0804
	Infection	0.5574	1	140.2	F (1, 53) = 0.3275	0.5695
	Diabetes x Sex	0.5453	1	137.1	F (1, 53) = 0.3204	0.5737
	Diabetes x Infection	1.288	1	324	F (1, 53) = 0.7570	0.3882
	Sex x Infection	0.5771	1	145.1	F (1, 53) = 0.3391	0.5628
	Diabetes x Sex x Infection	0.08441	1	21.23	F (1, 53) = 0.04960	0.8246
	Residual		53	428		
**Kidney T Cells**	**Diabetes**	**16.78**	**1**	**759.3**	**F (1, 53) = 11.92**	**0.0011**
	Sex	0.4224	1	19.12	F (1, 53) = 0.3002	0.5861
	Infection	0.000142	1	0.00642	F (1, 53) = 0.0001008	0.992
	Diabetes x Sex	0.1219	1	5.519	F (1, 53) = 0.08665	0.7696
	**Diabetes x Infection**	**7.407**	**1**	**335.3**	**F (1, 53) = 5.264**	**0.0258**
	Sex x Infection	1.26	1	57.04	F (1, 53) = 0.8956	0.3482
	Diabetes x Sex x Infection	0.1146	1	5.188	F (1, 53) = 0.08146	0.7764
	Residual		53	63.69		

Table S2: Three-way ANOVA results for flow cytometry data. Rows are bolded where P<0.05.
