# Supplementary material for: Diabetes Mellitus Affects Urinary Tissue Tropism of Group B Streptococci in a Sex-Dependent Manner
Source: Pathogens. 2026 Sep 1;15(9):923. doi: 10.3390/pathogens15090923 (PMC13610892; doi:10.3390/pathogens15090923)
Supplement: Supplementary file 1 [file pathogens-15-00923-s001.zip › pathogens-4407734-supplementary final 2 - update/Table S3.pdf]

	Source of Variation	% of Total Variation	DF	MS	F (DFn, DFd)	P value
**Bladder CXCL1**	**Diabetes**	**22.15**	**1**	**12137**	**F (1, 27) = 26.52**	**<0.0001**
	**Sex**	**45.4**	**1**	**24872**	**F (1, 27) = 54.35**	**<0.0001**
	**Interaction**	**22.76**	**1**	**12466**	**F (1, 27) = 27.24**	**<0.0001**
	Residual		27	457.6		
**Bladder CCL2**	**Diabetes**	**18.44**	**1**	**203.4**	**F (1, 26) = 6.888**	**0.0143**
	Sex	3.313	1	36.55	F (1, 26) = 1.238	0.2761
	Interaction	6.257	1	69.04	F (1, 26) = 2.338	0.1384
	Residual		26	29.53		
**Kidney CCL3**	**Diabetes**	**19.37**	**1**	**113.9**	**F (1, 25) = 6.261**	**0.0192**
	Sex	2.083	1	12.24	F (1, 25) = 0.6730	0.4198
	Interaction	0.9411	1	5.533	F (1, 25) = 0.3041	0.5862
	Residual		25	18.19		
**Kidney IL-1b**	**Diabetes**	**28.39**	**1**	**708.1**	**F (1, 25) = 10.44**	**0.0034**
	Sex	4.282	1	106.8	F (1, 25) = 1.575	0.2211
	Interaction	0.8184	1	20.42	F (1, 25) = 0.3010	0.5881
	Residual		25	67.83		
**Kidney IL-17**	**Diabetes**	**16.47**	**1**	**0.6445**	**F (1, 26) = 1.690**	**0.0156**
	**Sex**	**19.86**	**1**	**2.553**	**F (1, 26) = 6.694**	**0.0086**
	Interaction	4.159	1	3.078	F (1, 26) = 8.071	0.205
	Residual		26	0.3814		

Table S3: Two-way ANOVA results for ELISA data. Rows are bolded where *P* <0.05.
